# Cardiopulmonary work up of patients with and without fatigue 6 months after COVID-19

**DOI:** 10.1038/s41598-022-22876-9

**Published:** 2022-10-27

**Authors:** Kirsten Thiele, Paul Balfanz, Tobias Müller, Bojan Hartmann, Jens Spiesshoefer, Julian Grebe, Dirk Müller-Wieland, Nikolaus Marx, Michael Dreher, Ayham Daher

**Affiliations:** 1 Department of Cardiology, Angiology and Intensive Care Medicine, University Hospital RWTH, 52074 Aachen, Germany; 2 Department of Pneumology and Intensive Care Medicine, University Hospital RWTH, 52074 Aachen, Germany

**Keywords:** Fatigue, Respiratory signs and symptoms

## Abstract

The pathogenesis of long-Covid symptoms remains incompletely understood. Therefore, we aimed to determine cardiopulmonary limitations 6 months after surviving COVID-19 using pulmonary function tests, echocardiographic studies to the point of analysis of global-longitudinal-strain (GLS), which describes the cycling myocardium deformation and provides better data on left ventricular (LV) dysfunction than LV ejection fraction (LVEF), and validated questionnaires. Overall, 60 consecutive hospitalized patients were included (61 ± 2 years, 40% treated in the ICU). At follow-up (194 ± 3 days after discharge), fatigue was the most prevalent symptom (28%). Patients with fatigue were more symptomatic overall and characterized by worse quality of life (QoL) scores compared to patients without fatigue (all *p* < 0.05), mainly due to limited mobility and high symptom burden. While PFT variables and LVEF were normal in the vast majority of patients (LVEF = 52% (45–52%)), GLS was significantly reduced (− 15% (− 18 to − 14%)). However, GLS values were not different between patients with and without fatigue. In conclusion, fatigue was the most prevalent long-Covid symptom in our cohort, which was associated with worse QoL mainly due to limited mobility and the high burden of concomitant symptoms. Patients showed a subtle myocardial dysfunction 6 months after surviving COVID-19, but this did not relate to the presence of fatigue.

## Introduction

As almost 2 years have passed since the beginning of coronavirus disease 2019 (COVID-19) pandemic, a growing population of individuals has recovered from severe acute respiratory syndrome coronavirus type 2 (SARS-CoV-2) infection. However, a growing body of evidence suggests that these patients may experience a wide range of physical, cognitive and mental symptoms after recovery from acute illness including but not limited to pain, depression, anxiety, fatigue and self-care issues which may persist for more than several months^1^. Symptoms that develop during or after COVID-19, continue for ≥ 2 months (i.e., 3 months from the onset of illness), have an impact on the patient's life, and are not explained by an alternative diagnosis are now referred to as “post-COVID conditions”^2,3^, "long-Covid" or "post-acute sequelae of SARS-CoV-2 infection (PASC)”^4^. Nevertheless, while the pathogenesis of long-Covid related symptoms such as fatigue is still incompletely understood, there is an agreement that these patients require a multidisciplinary physical and psychological approach with a careful symptom evaluation by means of standardized questionnaires, but also with functional examinations including cardiopulmonary assessment^5^. While data of follow-up pulmonary function tests (PFTs) is evolving, less data is available regarding cardiac function in patients with long-Covid^4^. One study reported that among patients admitted to the intensive care unit (ICU) due to COVID-19 who were still symptomatic 4 months after discharge, 10% had a left ventricular ejection fraction (LVEF) of less than 50%^6^. Therefore, further assessment of left ventricular (LV) function in patients with long-Covid using additional modalities such as global longitudinal strain (GLS) analysis by speckle-tracking echocardiography might provide explanations for long-Covid. GLS describes the cycling myocardium deformation (shortening or lengthening), and is known to provide better information on LV dysfunction than LVEF yet yielding additional prognostic information^7^. Concerning COVID-19, GLS was shown to be reduced in a significant proportion of patients during their acute infection, even in those with normal LVEF; but more importantly it seems to predict clinical outcomes in these patients^8–12^, and subclinical LV dysfunction detected by GLS seems to persist in nearly a third of recovered COVID-19 patients one month after recovery despite normal LVEF^13,14^. However, long-term studies are needed to better understand the cardiopulmonary limitations, including LV dysfunction, after COVID-19 and to assess their clinical implications and progression over time.

The main aim of the current study was to determine physical and psychological symptoms by means of validated questionnaires, but also to determine cardiopulmonary limitations using advanced work-up including GLS in COVID-19 survivors, 6 months after discharge from the hospital. These data could provide a better understanding of this emerging disease and could help to ensure an adequate and timely management of significant health limitations in an attempt to restore premorbid quality of life (QoL)^15^.

## Materials and methods

The present prospective study included 60 consecutive patients who had been hospitalized due to COVID-19 confirmed by reverse-transcriptase–polymerase-chain-reaction (RT-PCR) in a respiratory tract sample. Patients were hospitalized if they had severe dyspnea with signs of respiratory decompensation such as increased respiratory rate, a new need for oxygen therapy (i.e. oxygen saturation (SpO2) ≤ 90% in patients without prior respiratory failure), or signs of acute organ dysfunction (e.g. altered mentation, acute renal failure). At discharge, patients got routine follow-up appointments in the pulmonary disease outpatient clinic of our institution.

The study protocol was approved by the local ethics committee (The Independent Ethics Committee at the RWTH Aachen Faculty of Medicine, EK 080/20). All investigations were performed in accordance with the ethical standards declared in the Declaration of Helsinki in its latest revision. Written informed consent was obtained from all patients, their legal representative in cases of severe consciousness disorders, or the consulting physician, if appropriate. Written informed consent was obtained from all patients as early as possible.

Regarding assessments performed during the hospital stay, demographic data, disease history, coexisting medical conditions, presence of chronic respiratory failure, smoking history, and medication history were recorded for all patients. Symptoms on admission and a detailed history of present symptoms were also documented. Patients were assessed for eligibility on the basis of a positive RT-PCR assay for SARS-CoV-2 in a respiratory tract sample. Serum, plasma, and whole blood samples were obtained routinely at the time of admission.

Concerning assessments performed at six-month follow-up, fatigue was identified as a symptom by means of a standardized clinical interview that comprised asking for physical or mental exhaustion or a reduced drive in pursuing activities of daily life. Physicians and study nurses were specifically trained to do this in a standardized fashion. Patients were classified as suffering from fatigue if they described difficulty or inability to start or maintain an activity (subjective feeling of weakness and loss of energy at rest or easy fatigability after starting an activity) leading to a marked decrease in motivation to pursue daily, routine activities, with or without difficulties in concentration, memory, and emotional stability. Full PFTs, electrocardiography and transthoracic echocardiography (TTE) were performed. TTE examinations were performed using commercially available ultrasound systems (GE Vingmed Ultrasound, Horten, Norway) and the echocardiographic measurements were obtained by a cardiologist blinded to all clinical information, in accordance with the guidelines of the EACI (European Association of Cardiovascular Imaging) and ASE (American Society of Echocardiography). Left ventricular systolic function (LVEF) was measured in 4 chamber and 2 chamber views according to Simpson’s Biplane Method. Additionally, we performed a myocardial deformation analysis of the left ventricle to assess peak global longitudinal strain (GLS) of the myocardium by speckle-tracking echocardiography in 4 chamber-, 2 chamber- and apical 3 chamber- views. Images were stored digitally for subsequent offline analysis. According to the latest American Echocardiography Association guidelines, GLS values >  − 16% was defined as diminished^16^.

Furthermore, blood samples were taken and health-related QoL was assessed. With support of a trained study team, patients completed different clinical questionnaires to assess various aspects of their QoL including: Patient Health Questionnaire 9 (PHQ-9) of depression^17^, Generalized Anxiety Disorder 7 (GAD-7) (on both scales, minimal symptoms are represented by a score of 0–4, mild symptoms by a score of 5–9, moderate symptoms by a score of 10–14 and severe symptoms by a score of ≥ 15)^18^, St. George’s Respiratory Questionnaire (SGRQ) (which is scaled from 0 representing optimal health to 100 reflecting worst health, and has three main components: symptoms component evaluates respiratory symptoms; activity component evaluates the physical activities; and the impact component assesses social and psychological limitations)^19,20^, and EQ-5D-5L (Euro Quality of life—five Dimensions—five Levels) questionnaire, which is a descriptive system that defines health in terms of 5 dimensions: Mobility, Self-Care, Usual Activities, Pain/Discomfort, and Anxiety/Depression^21^. Whole-body plethysmography (MasterLab; Viasys, Hoechberg, Germany) was performed before and after bronchodilation (including diffusing capacity for carbon monoxide (DLco) measurement only after bronchodilation) according to current guidelines and recommendations^22–24^. Samples for blood gas analyses (BGA) were taken from the arterialized earlobes of all patients while breathing room air without supplemental oxygen (ABL 800 flex; Radiometer, Copenhagen, Denmark). All patients underwent a 6-min walk test (6MWT) without supplemental oxygen, with measurements of vital signs including SpO_2_ and Borg-scale before and after exercise according to current recommendations^25–27^.

Statistical analyses were performed using standard descriptive statistics including mean ± standard deviation, median (interquartile range), frequencies and percentages (%). Analyses were performed in patient subgroups (patients with fatigue at follow-up versus those without fatigue). Between-group differences were tested using Two-way-ANOVA test and χ^2^ test for continuous and categorical variables, respectively. Nominal *p* values are presented. To eliminate the effects of potential confounders, we repeated the analyses after excluding patients with previous cardiac comorbidities. Furthermore, we used a multivariate logistic regression in three generalized linear models (R version 4.1.2) to test our univariate analysis for clinically determined confounders. The response variable in all models was self-reported fatigue at six-month follow-up visit. The first model included the following variables: Age, sex, forced expiratory volume in 1s (FEV_1_), vital capacity (VC), GLS, the presence of a previous respiratory disease, heart disease, chronic kidney disease, diabetes mellitus, overweight, obesity, malignancy and hepatitis. The second model included variables that may relate to cardiac function: Age, sex, GLS, previous heart disease, diabetes mellitus, overweight, obesity, admission to ICU during acute infection and CRP level on hospital admission. The third model included variables that may relate to PFTs: previous respiratory disease, admission to ICU, CRP on hospital admission, FEV1, VC, DLco/VA and distance in 6MWT. Nominal p values are presented.

## Results

60 patients (age 61 ± 2, 67% male) were included in this analysis. Baseline characteristics and variables during hospital stay, including laboratory parameters and disease severity, as well as comorbidities of the study population are described in Table 1.

**Table 1:** Baseline characteristics and comorbidities of the study population.

	Total (n = 60)	Fatigue (n = 17)	No fatigue (n = 43)	*p* value
**Characteristics**				
Age, years	61 ± 2	59 ± 4	61 ± 2	0.38
Female	20 (33)	9 (53)	11 (26)	0.06
Male	40 (67)	8 (47)	32 (74)	0.06
ARDS	18 (30)	4 (24)	14 (33)	0.55
**Symptom onset to, days**				
Hospitalization	7 ± 0	8 ± 1.7	7 ± 0.8	0.25
ICU admission	10 ± 0.6	13 ± 2.2	9 ± 0.7	0.60
**Inpatient management**				
ICU	24 (40)	4 (24)	20 (47)	0.14
Invasive ventilation	18 (30)	4 (24)	13 (30)	0.75
Extracorporeal membrane oxygenation	3 (5)	0 (0)	3 (7)	0.55
Prone positioning	14 (23)	2 (12)	11 (26)	0.31
Dialysis	8 (13)	2 (12)	5 (12)	> 0.99
Antibiotic therapy	23 (38)	5 (29)	16 (37)	0.76
**Periods, days**				
Fever days	16 ± 2.1	16 ± 3	17 ± 2.6	0.11
Hospitalization	25 ± 2.6	22 ± 4.8	27 ± 3.4	0.16
ICU length of stay	32 ± 2.9	36 ± 3.2	31 ± 3.7	0.58
**Laboratory findings on admission**				
D-dimer [ng/ml]	1418 [890–4952]	1128 [983–1273]	2169 [957–6103]	0.18
LDH [U/l]	378 [290–458]	330 [268–445]	402 [295–458]	0.82
CK [U/l]	124 [63–327]	107 [57–297]	124 [67–351]	0.53
CK_MB_ [U/l]	19 [16–25]	17 [16–19]	22 [15–27]	0.69
hs-Troponin T [pg/ml]	13 [6–19]	11 [4–19]	13 [7–18]	0.80
Creatinine [mg/dl]	1 [0.8–1.2]	0.9 [0.9–1.2]	1 [0.8–1.3]	0.96
CRP [mg/l]	81 [44–153]	45 [26–107]	94[54–159]	0.69
PCT [ng/ml]	0.1 [0.1–0.3]	0.1 [0.1–0.1]	0.2 [0.1–0.4]	0.32
Ferritin [ng/ml]	1185 [760–2046]	799 [528–1069]	1185 [771–2364]	0.25
**Comorbidities**				
Total	53 (88)	16 (94)	37 (86)	0.66
Arterial hypertension	31 (52)	1 (6)	3 (7)	> 0.99
COPD	8 (13)	3 (18)	5 (12)	0.68
Bronchial asthma	8 (13)	2 (12)	6 (14)	> 0.99
Pre-existing heart diseases	13 (22)	2 (12)	6 (14)	> 0.99
Overweight (BMI ≥ 25 kg/m^2^, < 30 kg/m^2^)	16 (27)	4 (24)	12 (28)	> 0.99
Obesity (BMI ≥ 30 kg/m^2^)	22 (37)	7 (41)	15 (35)	0.76
Chronic kidney disease	9 (15)	2 (12)	5 (12)	> 0.99
Smoking				
Former smoking	10 (17)	5 (41)	5 (12)	0.26
Current smoking	9 (15)	3 (18)	6 (14)	0.70
Diabetes mellitus	16 (27)	4 (24)	5 (12)	0.25
Malignancy	12 (20)	4 (24)	8 (19)	0.72
Chronic hepatitis	7 (12)	2 (12)	5 (12)	> 0.99
Peripheral arterial occlusive disease	4 (7)	1 (6)	3 (7)	> 0.99

Values are presented as mean ± standard deviation, number of patients (percentage) or median [interquartile range].

ARDS, Acute Respiratory Distress Syndrome; ICU, Intensive Care Unit; LDH, lactate dehydrogenase; CK, creatine kinase; hs-Troponin T, high sensitive troponin-T; CRP, C-reactive protein; PCT, Procalcitonin; COPD, chronic obstructive pulmonary disease; BMI, Body Mass Index.

Patients were seen in our pulmonary disease outpatient clinic for a follow-up examination 6 months after discharge (time to follow-up 194 ± 2.5 days). Table 2 shows symptoms of patients at follow-up visit. The most prevalent symptom was fatigue (28%). Patients with fatigue experienced significantly more other symptoms than those without fatigue, including headache (*p* < 0.001), myalgia (*p* = 0.007), dyspnea (*p* = 0.02), chest pain (*p* = 0.004), nausea (*p* = 0.004) and cognitive disorders (*p* = 0.001). Consistently, patients with symptoms of fatigue were more likely to complain of more limitations in their QoL than those without fatigue, especially due to higher symptom burden (*p* < 0.001) and reduced mobility (*p* < 0.001). Patients with fatigue showed worse depression and anxiety scores than patients without fatigue (*p* < 0.001), however, at the same time, all patients had at most mild to moderate symptoms in these two categories (Table 2).

**Table 2:** Follow-up parameters at 6-month time-point.

	Total (n = 60)	Fatigue (n = 17)	No fatigue (n = 43)	p value
Height [cm]	173 [166–178]	165 [158–176]	174 [168–179]	0.12
Weight [kg]	86 [79–98]	90 [70–102]	85 [80–96]	0.73
BMI [kg/m^2^]	29 [26–31]	29 [25–41]	29 [26–31]	0.51
Respiratory rate [bpm]	17 [16–20]	17 [16–20]	17 [16–19]	0.35
Oxygen saturation [%]	98 [97–98]	97 [96–98]	98 [97–99]	0.79
Oxygen flow [l/min]	0 [0–0]	0 [0–0]	0 [0–0]	0.59
Temperature [°C]	37 [36, 37]	37 [36, 37]	37 [36, 37]	0.87
BP_sys_ [mmHg]	136 [125–150]	135 [118–142]	140 [125–150]	0.39
BP_dia_ [mmHg]	92 [80–100]	89 [77–97]	92 [82–102]	0.52
Heart rate [bpm]	80 [69–90]	80 [73–80]	78 [66–91]	0.28
Frailty Score	3 [2–5]	3 [3–6]	3 [2, 3]	0.02
**Laboratory findings**				
D-dimer [ng/ml]	356 [268–544]	425 [268–544]	313 [241–489]	0.96
LDH [U/l]	201 [180–236]	208 [189–236]	192 [174–230]	0.59
CK [U/l]	88 [71–123]	87 [80–98]	86 [65–142]	0.01
CK_MB_ [U/l]	2.8 [1.9–3]	2.4 [1.7–2.9]	2.9 [1.9–3.1]	0.84
hs-Troponin T [pg/ml]	8.5 [6–15]	6 [4–14]	9 [6–15]	0.23
NT_pro_BNP [pg/ml]	90 [29–258]	77.6 [31–203]	84.8 [25–297]	0.05
Creatinine [mg/dl]	1 [0.8–1.1]	1 [0.8–1.1]	1 [0.8–1.1]	0.74
CRP [mg/l]	1.9 [1–4, 7]	2.6 [1, 6, 7]	1.6 [0.9–2.8]	0.04
PCT [ng/ml]	0.1 [0–0.1]	0.1 [0–0.1]	0.1 [0–0.1]	0.99
Ferritin [ng/ml]	156 [60–310]	168.3 [54–360]	146.5 [60–279]	0.43
**Symptoms**				
Fatigue	17 (28)	17 (100)	0 (0)	< 0.001
Dyspnea	15 (25)	8 (47)	7 (16)	0.02
Cough	8 (13)	4 (24)	4 (9)	0.20
Headache	16 (27)	10 (59)	6 (14)	< 0.001
Myalgia	11 (18)	7 (41)	4 (9)	0.007
Rhinorrhea	6 (10)	3 (18)	3 (7)	0.33
Chest pain	8 (13)	6 (35)	2 (5)	0.004
Cognitive disorders	3 (5)	3 (18)	0 (0)	0.01
Loss of taste	5 (8)	3 (18)	2 (5)	0.13
Loss of smell	6 (10)	1 (6)	5 (12)	0.66
Sore throat	5 (8)	5 (29)	0 (0)	0.001
Nausea	4 (7)	4 (24)	0 (0)	0.004
Emesis	3 (5)	3 (18)	0 (0)	0.01
**Questionnaires at follow up**				
PHQ-9	5 [2–8]	11 [7–16]	3 [1–6]	< 0.001
GAD-7	3 [0–7, 25]	8 [6–14]	1 [0–4]	< 0.001
SGRQ				
Symptoms Score	17.6 [3–43]	45.4 [2–64]	8.7 [0–22]	< 0.001
Activity Score	41.2 [3–77]	79.8 [1, 7–86]	9.4 [0–47]	< 0.001
Impacts Score	5.2 [0–29]	31.3 [7–44]	0 [0–5]	0.001
Total Score	13.1 [1, 6–45]	50.2 [1–63]	3.9 [0, 3–12]	0.002
EQ-5D-5L				
Mobility/Walking score	1 (1–3)	2 (1–4)	1 (1–2)	0.001
Self-Care score	1 (1–2)	1.5 (1–3)	1 (1–1)	0.13
Usual Activities score	1 (1–3)	3 (2–3)	1 (1–1)	< 0.001
Pain/Discomfort score	2 (1–3)	2.5 (2–4)	1 (1–2)	0.01
Anxiety/Depression score	1 (1–2)	1 (1–2.3)	1 (1–1)	0.13
**6MWT**				
Distance, m	443 [359–521]	390 [290–440]	470 [363–525]	0.31
SpO_2_ before exercise, %	97 [95–98]	96 [95–98]	97 [95–98]	0.45
SpO_2_ after exercise, %	97 [95–98]	97 [95–98]	97 [95–98]	0.16
HR before exercise, bpm	78 [67–90]	73 [67–77]	80 [69–91]	0.34
HR after exercise, bpm	90 [80–107]	89 [73–108]	91 [82–106]	0.94
Dyspnea on Borg scale before exercise	0 [0–2]	2 [0–3]	0 [0–1]	0.01
Dyspnea on Borg scale after exercise	2 [0–3]	3 [0–5]	1 [0–3]	0.05
Fatigue on Borg scale before exercise	0 [0–2]	2 [0–4]	0 [0–1]	0.06
Fatigue on Borg scale after exercise	2 [0–3]	4 [2–6]	2 [0–3]	0.02
**Pulmonary function tests**				
TLC [%]	98 [85–107]	101 [9–115]	98 [85–105]	0.33
VC [%]	94 [81–103]	96 [81–102]	93 [78–103]	0.14
FVC [%]	93 [80–104]	96 [83–105]	92 [76–101]	0.15
RV [%]	111 [96–134]	123 [89–146]	105 [97–126]	0.47
RV/TLC [%]	109 [97–121]	117 [100–128]	108 [96–118]	0.17
FEV_1_ [%]	95 [81–106]	86 [67–95]	97 [84–110]	0.03
FEV_1_/FVC [%]	81 [77–86]	78 [72–81]	84 [78–88]	0.002
R_eff_ [%]	91 [68–114]	100 [87–122]	85 [66–104]	0.16
DL_CO_ [%]	69 [55–77]	63 [45–71]	69 [55–78]	0.38
DL_CO_/VA [%]	84 [75–95]	78 [68–83]	86 [77–97]	0.18
PaO_2_ [mmHg]	73 [65–83]	66 [58–78]	76 [66–84]	0.12
PaCO_2_ [mmHg]	38 [34–39]	38 [32–40]	37 [34–39]	0.52
pH	7.4 [4–7]	7.42 [7.41–7.5]	7.4 [4–7]	0.87
Base excess [mmol/l]	0.6 [− 0.4–1.3]	0.7 [0.15–1.3]	0.4 [− 0.8–1.2]	0.29
**Echocardiography**				
LVEF—global normal	49 (82)	15 (88)	34 (79)	0.70
LVEF [%]	52 [45–52]	49 [47–50]	52 [46–53]	0.75
RVEF global normal	53 (88)	15 (88)	38 (88)	> 0.99
TAPSE	21 [18–23]	22 [20–23]	20 [18–23]	0.27
RVSP + CVP [mmHg]	26 [24–32]	28 [25–30]	26 [23–32]	0.84
GLS > − 16, n (%)	41 (68)	9 (53)	32 (74)	0.09
GLS [%]	− 15 [− 18–14]	− 17 [− 20–15]	− 15 [− 18–14]	0.34

Values are presented as mean ± standard deviation, number of patients (percentage) or median [interquartile range].

BMI, body-mass-index; BP, blood pressure; LDH, lactate dehydrogenase; CK, creatine kinase; hs-Troponin T, high sensitive troponin-T; NT_pro_BNP, N-terminal pro B-type natriuretic peptide; CRP, C-reactive protein; PCT, Procalcitonin; PHQ-9, Patient Health Questionnaire 9; GAD-7, Generalized Anxiety Disorder 7; SGRQ, St. Georges’s Respiratory Questionnaire; EQ-5D-5L, Euro Quality of Life-Five Dimensions-Five Levels; 6MWT, six- minute walk test; HR, heart rate; TLC, total lung capacity; VC, vital capacity; RV, residual volume; FEV_1_, forced expiratory volume in 1s; FVC, forced vital capacity; R_eff_, effective specific resistance; DL_co_, diffusing capacity for carbon monoxide; VA, alveolar volume; PaO_2_, partial pressure of oxygen; PaCO_2_, partial pressure of carbon dioxide; LVEF, left ventricular ejection fraction; RVEF, right ventricular ejection fraction; TAPSE, tricuspid annular plane systolic excursion; RVSP, right ventricular systolic pressure; CVP, central venous pressure; GLS, global longitudinal strain.

Regarding whole-body plethysmography values and BGA, all patients revealed no impairments independent of the presence of fatigue (Table 2). Although PFT variables were normal in both groups, patients with fatigue showed lower values for FEV1 than those without fatigue in the univariate analysis: (FEV1: 86% (67–95%) vs. 97% (84–110%), respectively; *p* = 0.03).

Regarding echocardiography, the mean LVEF of all patients was normal (median: 52% (Interquartile range (IQR): 45–52%)), whereas LV myocardial deformation analysis revealed impaired GLS in the whole group at 6-month follow-up (median GLS: − 15% (− 18 to − 14%)). 41 patients (68%) had significant LV dysfunction with GLS values >  − 16% (Table 2). However, there were no significant differences in GLS between patients with and without fatigue (*p* = 0.34) (Table 2). In addition, when patients who had been treated in the ICU during the hospital stay were compared to patients who had been treated on the general ward no differences in LVEF (*p* = 0.21) or in GLS (*p* = 0.14) were found (Table 3).

**Table 3:** Comparison of follow-up TTE between patients treated in the ICU vs. general ward.

	Total (n = 60)	ICU (n = 19)	non-ICU (n = 41)	p value
LVEF—global normal	49 (82)	15 (79)	34 (83)	0.72
LVEF [%]	52 [45–52]	52 [49–58]	50 [46–52]	0.21
RVEF—global normal	53 (88)	16 (84)	37 (90)	0.66
TAPSE	21 [18–23]	21 [18–24]	20 [18–23]	0.89
RVSP + CVP, mmHg [mmHg]	26 [24–32]	25 [23–31]	27 [25–32]	0.97
GLS > − 16, n (%)	41 (68)	11 (58)	30 (73)	0.08
GLS [%]	− 15 [− 18 to − 14]	− 17 [− 18 to − 14]	− 15 [− 17 to − 14]	0.14

Values are presented as mean ± standard deviation, number of patients (percentage) or median [interquartile range].

LVEF = left ventricular ejection fraction; RVEF = right ventricular ejection fraction; TAPSE = tricuspid annular plane systolic excursion; RVSP = right ventricular systolic pressure; CVP = central venous pressure; GLS = global longitudinal strain.

In our first multivariate regression model (Table 4), fatigue was more prevalent among female patients but with a very wide confidence interval (Odds ratio (OR) 8.325 [95% CI: 1.21–100.10], *p* < 0.05). Other variables including comorbidities, age and overweight/obesity were comparable between both groups (all *p* > 0.05). Although GLS values was worse in patients with fatigue than those without fatigue after excluding patients with history of cardiac disease (supplementary Table S1), both GLS and PFTs (represented in FEV_1_ and VC) were comparable in patients with and without fatigue in the multivariate models.

**Table 4:** Multivariate logistic regression at 6-month follow-up.

	OR [95% CI]	*p* value
**Model 1**		
Age, years	0.943 [0.84–1.03]	0.23
Female sex	8.325 [1.21–100.10]	< 0.05
Respiratory disease	3.49 [0.48–33.39]	0.23
Chronic kidney disease	0.03 [0.00–0.93]	0.09
Heart disease	22.58 [0.92–1431.38]	0.08
Diabetes mellitus	2.42 [0.23–27.40]	0.45
Overweight (BMI ≥ 25 kg/m^2^, < 30 kg/m^2^)	0.31 [0.02–2.83]	0.32
Obesity (BMI ≥ 30 kg/m^2^)	2.20 [0.20–35.70]	0.53
Malignancy	4.44 [0.14–214.49]	0.40
Chronic hepatitis	3.42 [0.06–230.88]	0.54
FEV_1_ [%]	0.93 [0.85–1.00]	0.06
VC [%]	1.08 [0.98–1.19]	0.12
GLS [%]	0.68 [0.42–0.98]	0.07
**Model 2**		
Age, years	0.96 [0.90–1.01]	0.16
Female sex	4.97 [0.98–31.98]	0.06
Heart disease	3.73 [0.49–31.15]	0.20
Diabetes mellitus	0.28 [0.03–1.68]	0.19
Overweight (BMI ≥ 25 kg/m^2^, < 30 kg/m^2^)	0.50 [0.07–3.33]	0.48
Obesity (BMI ≥ 30 kg/m^2^)	1.31 [0.22–8.43]	0.77
Admission to ICU	0.58 [0.07–3.69]	0.58
CRP on hospital admission, [mg/l]	1.00 [0.98–1.01]	0.36
GLS [%]	0.79 [0.59–1.02]	0.09
**Model 3**		
Respiratory disease	0.38 [0.00–7.42]	0.58
Admission to ICU	1.17 [0.05–21.01]	0.91
CRP on hospital admission, [mg/l]	1.00 [0.99–1.02]	0.69
FEV1 [%]	0.97 [0.89–1.05]	0.39
VC [%]	1.03 [0.94–1.15]	0.50
DL_CO_/VA [%]	0.95 [0.85–1.04]	0.32
Distance in 6MWT, [meters]	1.00 [0.99–1.01]	0.85

Results from three multivariate logistic regressions, response variable is patient-reported fatigue at 6 months follow-up in each model. Regarding model 3: Pulmonary function tests in these models represent percentage of normal values (adjusted for age, gender, height and race). For numerical variables a one unit of change is associated with a change in likelihood of reporting fatigue at 6 months follow-up by the value reported in the OR column. OR > 1 increase the likelihood, while OR < 1 reduce the likelihood.

OR, Odds-Ratio; 95% CI, 95% confidence interval; BMI, Body mass index in kg/m^2^; FEV_1_, forced expiratory volume in 1s; VC, vital capacity; GLS, global longitudinal strain; CRP, C-reactive protein; DLco, diffusing capacity for carbon monoxide; VA, alveolar volume.

### Ethics approval and consent to participate

The study protocol was approved by the local ethics committee (The Independent Ethics Committee at the RWTH Aachen Faculty of Medicine, EK 080/20). All investigations were performed in accordance with the ethical standards laid down in the Declaration of Helsinki in its latest revision. Written informed consent was obtained from all patients, their legal representative in cases of severe consciousness disorders, or the consulting physician, if appropriate. Written informed consent was obtained from all patients as early as possible.

## Discussion

In the present study, fatigue was the most prevalent long-Covid symptom 6 months after recovery from acute COVID-19, which was accompanied with additional symptoms and significantly impaired QoL scores, mainly due to limited mobility and high symptom burden. In addition, patients showed subtle myocardial dysfunction detected by global longitudinal strain analysis 6 months after COVID-19, which did not correlate with the presence of fatigue. These abnormalities might be missed when left ventricular function is evaluated by LVEF only, as LVEF was not impaired in the majority of patients.

Fatigue is known to be among the most common persistent symptoms after COVID-19^28^, and although it is likely to improve over time, it may persist beyond 6 months^5,29^. Our results are in accordance with previous studies showing that fatigue is the most common symptom in patients with long-Covid^6,30,31^. Patients with fatigue were impaired mainly due to immobility, as reflected by worse SGRQ activity scores and EQ-5D-5L mobility and usual activity score values. In addition, patients with fatigue were characterized by a high symptom burden, as reflected by high SGRQ symptom scores, including pain symptoms such as headache, as well as gastrointestinal symptoms such as nausea and vomiting. Although patients with fatigue showed worse PHQ-9 and GAD-7 values, all patients suffered from at most mild to moderate anxiety and depression. These findings may help to further characterize patients with fatigue, as this symptom is often a self-reported condition without clearly defined diagnostic criteria, and using validated QoL questionnaires may help to identify patients with serious limitations who require further evaluation and management.

The pathogenesis of fatigue after COVID-19 infection is not fully understood yet and is likely to be multifactorial. In our study, patients with and without fatigue at follow-up showed comparable characteristics during their acute disease which comprised the need for ICU management, length of ICU stay, laboratory findings and comorbidities. However, the “multidisciplinary collaborative consensus guidance statement on the assessment and treatment of fatigue in post-acute sequelae of SARS‐CoV‐2 infection (PASC) patients” recommended a multidisciplinary assessment including exercise and cardiopulmonary testing in patients with long-Covid fatigue^5^. While 45% of COVID-19 patients show a performance below the lower limit of normal in the 6-min walk test 6 weeks after infection, more than 20% of patients would still have such limitations 6 months thereafter regardless of severity of the acute illness^30,32^. Although the pathogenesis of these limitations is likely multifactorial (muscular, cardiopulmonary, psychological …., etc.)^33^, cardiopulmonary evaluation is always required in patients with impaired exercise capacity. However, data on cardiac function in patients after COVID-19 infection is sparse and just evolving. Small studies showed short-term subtle myocardial dysfunction along with myocardial edema and fibrosis in patients recovering from COVID-19, even in young athletes^34–36^; and some preliminary data suggested that this myocardial dysfunction may persist over a longer period^37^. In previous studies, about 90% of patients who survived severe COVID-19 on the National Institute of Health (NIH) severity scale still had significant GLS impairments one month after recovery from the acute disease^13,38^, and some data suggests that GLS abnormalities could persist to longer periods even in patients who had mild disease during acute infection^39^. In our study, we not only used conventional analyses of cardiac function such as LVEF but also examined GLS of left ventricular myocardium using speckle-tracking echocardiography 6 months after recovery from severe COVID-19 on NIH severity scale. Interestingly, the vast majority of patients in our cohort showed normal cardiac function when conventional modalities (e.g. LVEF) were used. However, most patients (68%) showed significantly reduced GLS. This could represent residual myocardial damage due to COVID-19 or may be the result of preexisting undiagnosed myocardial dysfunction. However, cardiac remodeling has been reported to occur after myocardial injury caused by viral infection^37,40^. Even among children, subclinical myocardial dysfunction seems to be still detectable after 6 months of follow-up after COVID-19 in a not negligible proportion of patients^41^. In the preliminary follow-up study by Wu et al., myocardial fibrosis was detected 6 months after recovery from COVID-19 by cardiac magnetic resonance imaging, even in patients with normal LVEF and without concomitant cardiac disease or preexisting conditions^37^. SARS-CoV-2 binds to the angiotensin-converting enzyme 2 receptor, which is located on the surface of host cells and is highly expressed in the heart^42,43^, which may facilitate a direct damage to myocardial cells^42,43^. Furthermore, endomyocardial biopsy performed in the short period after recovery from COVID-19 showed an active inflammatory infiltrate^43–45^. Against this background, our results suggest that analyzing GLS in COVID-19 recoverees may help to detect subtle myocardial dysfunction even if LVEF is normal. The question as to whether these GLS abnormalities affect future outcomes and prognosis in COVID-19 survivors should be addressed by future studies, taking into account the known prognostic significance of GLS. However, the presence of fatigue did not correlate with GLS impairments and other echocardiographic variables in our cohort, a result that remained valid even after excluding patients with previous cardiac comorbidities and after adjustment for clinically determined confounders in the multivariable logistic regression analysis. Overall, subtle myocardial dysfunction, which is common 6 months after COVID-19, could not explain fatigue in our study. Moreover, the severity of COVID-19, expressed as the need of ICU management, did not correlate with worse GLS at follow-up.

Many previous studies have shown that lung function tends to normalize in patients recovering from COVID-19^30^, even in those who experienced a severe disease and required to be mechanically ventilated^31^. Our data also confirmed these results in both groups of patients, with and without fatigue. Nevertheless, patients with fatigue tended to have lower dynamic lung volumes in the univariate analysis. This finding is supposed to be of minor clinical importance since values were still in the normal range and the difference did not remain significant in the multivariate analyses. We showed recently in a preliminary analysis that patients with long-Covid show diaphragmatic dysfunction which was associated with more symptoms (mainly dyspnea) 1 year after acute illness^46^, and a recent study including COVID-19 survivors who underwent cardiopulmonary exercise testing 3 months after discharge, reported that functional limitations were present in one third of the subjects and were mainly explained by muscular impairment^47^. Whether fatigue after COVID-19 is related to chronic respiratory muscle dysfunction is an interesting question for future larger studies.

Our study has some limitations that need to be addressed. First, there were more than twice as many participants in the fatigue group compared to the non-fatigue group, which may have influenced the results. Second, the number of patients studied was quite small. Nevertheless, this preliminary data could form the basis for future studies aimed at better understanding symptoms and functional limitations after COVID-19. Third, the validity and reliability of the SGRQ for COVID-19 has not yet been assessed. Lastly, a more in-depth evaluation to detect myocardial dysfunction, e.g. by cardiac magnetic resonance imaging, could complete our obtained results and the absence of radiological examinations does not allow a conclusion on interstitial lung changes including pulmonary fibrosis in our cohort. Such examinations could be part of further analyses on this topic.

## Conclusions

Six months after hospital discharge, fatigue was the most common symptom after COVID-19 in our cohort, with a prevalence of 28%. Fatigue was accompanied by worse QoL scores mainly due to limited mobility and high burden of concomitant symptoms. In addition, using advanced echocardiography with strain analysis, we detected myocardial dysfunction that did not correlate with the presence of fatigue.

## Supplementary Information

Supplementary Information.

## Data Availability

The raw data that support the findings of this study are available from the Clinical Study Center (KKS) of the Clinic for Cardiology, Angiology and Intensive Care Medicine and the Clinic for Pneumology and Intensive Care Medicine of RWTH Aachen University Hospital, but restrictions apply to the availability of these data, which were used under license for the current study, and so are not publicly available. Data are however available from the authors upon request and with permission of KKS.
