# Supplementary material for: Cardiopulmonary work up of patients with and without fatigue 6 months after COVID-19
Source: Sci Rep. 2022 Oct 27;12:18038. doi: 10.1038/s41598-022-22876-9 (PMC9607837; doi:10.1038/s41598-022-22876-9)
Supplement: Supplementary file 1 — Supplementary Information. [file 41598_2022_22876_MOESM1_ESM.docx]

**Table S1.** Follow up parameters at 6 month time-point without patients with cardiac comorbidities

|  | **Total (n=47)** | **Fatigue (n=14)** | **No Fatigue (n=33)** | **p-value** |
| --- | --- | --- | --- | --- |
| **Symptoms** |  |  |  |  |
| - Fatigue | 14 (30) | 14 (100) | 0 (0) | **<0.01** |
| - Dyspnea | 11 (23) | 6 (43) | 5 (15) | **0.03** |
| - Cough | 7 (15) | 3 (21) | 4 (12) | 0.53 |
| - Headache | 13 (28) | 8 (57) | 5 (15) | **0.01** |
| - Myalgia | 8 (17) | 6 (43) | 2 (6) | **<0.01** |
| - Rhinorrhoea | 5 (11) | 2 (14) | 3 (9) | 0.71 |
| - Chest pain | 7 (15) | 5 (36) | 2 (6) | **0.01** |
| - Cognitive disorders | 3 (6) | 3 (21) | 0 (0) | **<0.01** |
| - Loss of smell | 3 (6) | 2 (14) | 1 (3) | 0.18 |
| - Loss of taste | 4 (9) | 1 (7) | 3 (9) | 0.76 |
| - Sore throat | 5 (11) | 5 (36) | 0 (0) | **<0.01** |
| - Nausea | 4 (9) | 4 (29) | 0 (0) | **<0.01** |
| - Emesis | 3 (6) | 3 (21) | 0 (0) | **0.01** |
| **Questionnaires** |  |  |  |  |
| - PHQ-9 | 5 [2—10] | 11 [7—16] | 4 [1—7] | **<0.01** |
| - GAD-7 | 4 [1—8] | 10 [6—14] | 1 [0—5] | **<0.01** |
| - SGRQ |  |  |  |  |
| - Symptoms score | 20.3 [6.3—47.3] | 47.9 [35.1—65.3] | 11.0 [0—25.8] | **<0.01** |
| - Activity score | 44.8 [4.4—77.4] | 73.3 [41.6—88] | 23.3 [0—60.8] | **<0.01** |
| - Impacts score | 8.8 [0—29.2] | 28.8 [20.8—41.8] | 3.6 [0—10.7] | **<0.01** |
| - Total score | 16.3 [3.7—44.9] | 46.8 [24.7—61.4] | 7.0 [1—25.2] | **<0.01** |
| - EQ-5D-5L |  |  |  |  |
| - Mobility/Walking score | 1 (1—2) | 2 (1—4) | 1 (1—2) | **0.01** |
| - Self-Care score | 1 (1—1.6) | 1 (1—2.3) | 1 (1—1) | **0.03** |
| - Usual activities score | 1 (1—3) | 2.5 (1.9—3) | 1 (1—1) | **<0.01** |
| - Pain score | 2 (1—3) | 2 (2—4) | 2 (1—2) | **0.01** |
| - Anxiety/Depression score | 1 (1—2) | 1 (1—2) | 1 (1—2) | 0.37 |
| **6MWT** |  |  |  |  |
| - Distance [m] | 473 [395—537] | 398 [355—524] | 480 [426—554] | 0.11 |
| - SpO2 before exercise [%] | 97 [96—98] | 97 [96—98] | 98 [96—98] | 0.45 |
| - SpO2 after exercise [%] | 97 [96—98] | 97 [96—98] | 98 [95—98] | 0.72 |
| - HR before exercise [bpm] | 79 [71—91] | 75 [70—80] | 85 [72—98] | 0.23 |
| - HR after exercise [bpm] | 97 [85—113] | 92 [85—116] | 97 [85—109] | 0.97 |
| - Dyspnea on Borg scale before exercise | 0 [0—2] | 2 [1—3] | 0 [0—1] | **0.01** |
| - Dyspnea on Borg scale after exercise | 3 [0—4] | 4 [3—6] | 2 [0—3] | **0.01** |
| - Fatigue on Borg scale before exercise | 0 [0—2] | 2 [2—4] | 0 [0—1] | **<0.01** |
| - Fatigue on Borg scale after exercise | 2 [1—4] | 5 [2—6] | 2 [0—3] | **<0.01** |
| **Pulmonary function tests** |  |  |  |  |
| - TLC [%] | 99 [89—108] | 101 [92—116] | 98 [89—108] | 0.45 |
| - VC [%] | 96 [87—104] | 96 [84—102] | 95 [87—107] | 0.77 |
| - RV [%] | 118 [95—136] | 123 [92—147] | 108 [96—127] | 0.19 |
| - RV/TLC [%] | 110 [96—121] | 121 [99—141] | 104 [95—118] | 0.09 |
| - FEV1 [%] | 96 [85—110] | 86 [66—95] | 99 [88—111] | **0.01** |
| - FEV1/FVC [%] | 80 [77—86] | 78 [72—81] | 83 [78—87] | **0.02** |
| - Reff [%] | 93 [74—115] | 117 [89—139] | 85 [66—103] | **<0.01** |
| - DLco [%] | 71 [63—78] | 65 [46—71] | 74 [64—80] | 0.09 |
| - DLco/VA [%] | 86 [78—98] | 78 [72—94] | 87 [80—100] | 0.11 |
| - PaO2 [mmHg] | 76 [66—83] | 74 [63—83] | 76 [66—85] | 0.72 |
| - PaCO2 [mmHg] | 37 [34—39] | 36 [32—41] | 37 [34—39] | 0.82 |
| - pH | 7.4 [7.4—7.5] | 7.4 [7.4—7.5] | 7.4 [7.4—7.4] | 0.19 |
| - Base excess [mmol/l] | 0.8 [-0.4—1.4] | 1.2 [0.5—2.3] | 0.6 [-1.0—1.4] | 0.19 |
| **Echocardiography** |  |  |  |  |
| - LVEF - global normal | 40 (85) | 13 (93) | 27 (82) | 0.33 |
| - LVEF [%] | 52 [48—54] | 52 [52—52] | 52 [47—55] | 1.00 |
| - RVEF global normal | 42 (89) | 13 (93) | 29 (88) | 0.61 |
| - TAPSE | 21 [18—23] | 22 [20—24] | 21 [18—23] | 0.23 |
| - RVSP + CVP [mmHg] - GLS > -16, n (%) | 25 [21—29]  27 (57) | 28 [23—34]  5 (36) | 25 [20—27]  22 (67) | 0.29  0.05 |
| - GLS [%] | -15 [-18—-14] | -18 [-20—-15] | -15 [-18—-14] | **<0.01** |

Values are presented as mean ± standard deviation, number of patients (percentage) or median [interquartile range]. BMI = body-mass-index; BP = blood pressure; LDH = lactate dehydrogenase; CK = creatine kinase; hs-Troponin T = high sensitive troponin-T; NTproBNP = N-terminal pro B-type natriuretic peptide; CRP = C-reactive protein; PCT = Procalcitonin; PHQ-9 = Patient Health Questionnaire 9; GAD-7 = Generalized Anxiety Disorder 7; SGRQ = St. Georges’s Respiratory Questionnaire; EQ-5D-5L = Euro Quality of Life – Five Dimensions – Five Levels; 6MWT = six- minute walk test; HR = heart rate; TLC = total lung capacity; VC = vital capacity; RV = residual volume; FEV1 = forced expiratory volume in 1s; FVC = forced vital capacity; Reff = effective specific resistance; DLco = diffusing capacity for carbon monoxide; VA = alveolar volume; PaO2 = partial pressure of oxygen; PaCO2 = partial pressure of carbon dioxide; LVEF = left ventricular ejection fraction; RVEF = right ventricular ejection fraction; TAPSE = tricuspid annular plane systolic excursion; RVSP = right ventricular systolic pressure; CVP = central venous pressure; GLS = global longitudinal strain.
